# Study of cenesthesias and body image aberration in schizophrenia

**DOI:** 10.4103/0019-5545.55086

**Published:** 2009

**Authors:** Gaurav Rajender, Krishna Kanwal, Dinesh M. Rathore, Deepa Chaudhary

**Affiliations:** Department of Psychiatry, G.B. Pant Hospital and Maulana Azad Medical College, New Delhi, India; 1 Department of Psychiatry, S.M.S. Medical College, Jaipur, Rajasthan, India; 2 Department of Psychiatry, Institute of Mental Health and Hospital, Agra, U.P, India; 3 Resident, S.M.S. Medical College, Jaipur, Rajasthan, India

**Keywords:** Body image, cenesthesias, schizophrenia

## Abstract

**Background:**

Abnormal body sensations are reported frequently by schizophrenic patients. Cenesthesias are infrequently recognized and diagnosis of cenesthopathic schizophrenia is rarely made. There are very few studies regarding the same.

**Aims and Objectives:**

To assess cenesthesias and different aspects of body image aberration and their relationship with psychopathology in patients with paranoid schizophrenia.

**Materials and Methods:**

Seventy patients of paranoid schizophrenia meeting the inclusion and exclusion criteria were assessed with Positive and Negative Symptom Scale(PANSS) for psychopathology, Bonn Scale for Assessment of Basic Symptoms/Category D ‘Cenesthesias’ (BSABS), Image-Marking Procedure(IMP), alteration of body size and body cathexis. Assessments were made at baseline and after two weeks and assessed with SPSS 12.0.

**Results:**

The most commonly endorsed items on BSABS were depersonalization, motor weakness, abnormal pain, numbness and stiffness. Patients underestimated lower extremities and feelings of body size change which was positively correlated with PANSS-scores and improved on reassessment. Cenesthesias were positively correlated with disturbances of body concept and were present at onset in 40% and change form in 75.7%.

**Conclusions:**

Cenesthesias and body image aberrations are common in paranoid schizophrenia. They are present from onset in few, change form and improve on treatment. Cenesthesias and disturbances of body concept are correlated and body size is associated with other psychopathology.

## INTRODUCTION

These heterogeneous disturbances of body experience and abnormal sensations are characteristic and frequent accessory symptoms.[1]

We have a host of reports on these puzzling sensations, particularly from schizophrenic patients.[2]

Cenesthesias and abnormal body sensations in schizophrenic patients are not uncommon and have been reported frequently in the past.[1,2] The word ‘coenesthesia’ was coined by Christian Reil, however it was more than hundred years after introduction of terminology in France with Dupre and Camus[3] that the concept of disordered cenesthesias became known as ‘cenesthopathy’. According to them cenesthopathy was a symptom of mental illness and pathological body sensations. These have been variously recognized as ‘vital feelings’ (Wernicke), ‘awareness of body’(Jasper), leibgefuhlsstorungen (disturbances of bodily feelings, Glatzel) and applied in the German (Coenasthesie, Gemeingefuhl), French (sensibilite generale, cenesthesie) and Russian (cenesthopathies) literature.[4] The scarcity with which this descriptive phenomenological entity is recognized though common is probably because the experiences may be labeled as different phenomenology. Most notably, Huber[5] described a subtype called ‘cenesthetic schizophrenia’, and the term ‘cenesthopathic schizophrenia’ is included within the category ‘other schizophrenia’ (F20.8) in ICD-10 classification.[6] Diagnostic Statistical Manual DSM-IV[7] does not have a similar diagnostic category. In clinical practice, this diagnosis is rarely made and the defining characteristics as well as its clinical relevance remain unclear. It appears that the majority of patients with marked bodily sensations are currently diagnosed as suffering from paranoid schizophrenia, possibly because these phenomena are generally classified as somatic hallucinations or delusional perception in Anglo- American literature. In this study we aimed to study the characteristics of cenesthesias and different aspects of body image aberration and their relationship with psychopathology, in a sample of patients with paranoid schizophrenia.

## MATERIALS AND METHODS

The study sample consisted of both outpatients and recently admitted inpatients between 18 and 60 years of age and suffering from paranoid schizophrenia. Diagnosis was initially made by the OPD or emergency psychiatrist according to ICD-10, and was later confirmed by research team member. Many patients were old cases and had been evaluated over years at the hospital, their diagnosis was further confirmed and course details were noted from the records. Patients with a history of a serious physical illness, physical disability, substance harmful use or dependence or on any medication in the last 2 weeks were excluded. All subjects gave informed consent. Detailed physical and neurological examination was done. The socio demographic and clinical data and details of psychiatric history were obtained from the patient or from their medical records. The follow-up assessment was carried out after 2 weeks.

### The following instruments were applied:

1. Positive and Negative Symptom Scale (PANSS)[8] was used to assess psychopathology. PANSS developed by Kay *et al.*, has 30 items which rate the patients clinical profile along a seven point continuum (1 = absent, 7 = extreme).Alpha coefficient analysis has indicated high internal reliability and homogeneity among PANSS items, with coefficients ranging from 0.73 to 0.83 (*P* < .001) for each of the scales.
2. Bonn Scale for Assessment of Basic Symptoms/Category D ‘Cenesthesias’ (BSABS).[9] The BSABS was developed by Gross and Huber. Klosterkotter *et al.*,[10] re-validated the sub-syndrome ‘cenesthesias’ through a multivariate cluster analysis of the BSABS.
3. Image-Marking Procedure (IMP)[11] was used for recording segmental body size perception of lower extremities. Patients marked distances as estimated in response to a two-point tactile stimulus by the investigator. Body perception indices (BPI) were calculated according to the established formula: Perceived size/real size × 100. Priebe and Rohricht[12] recently described psychometric properties of this instrument, indicating good internal consistency: Cronback's alpha between 0.78 to 0.84.
4. Body cathexis was self-rated on a 10-cm long Visual Analogue Scale. Patients were asked “How satisfied are you with your body?” (rated from 0 = totally dissatisfied to 10 = totally satisfied).[13] Similarly three other aspects as ‘small’ (feeling as if the body or its parts is/are unusually small), ‘large’ (feeling as if the body or its parts is/are unusually large) and ‘alteration of body size’ (feeling as if the body size has changed) were also assessed with 0 = absolutely wrong and 10 = absolutely right.

Antipsychotic medication was recorded in chlorpromazine equivalents, calculated according to Kane.[14] The Montgomery Asberg Depression rating scale(MADRS) was used to assess symptoms of depression. Adverse effects of medication were documented as reported by the patient subjectively and were assessed using the Extrapyramidal Symptom Rating Scale (ESRS).[15]

To assess the association between cenesthesias, body image aberration and other psychopathology Pearson's correlation coefficients were calculated. The analysis was carried out using SPSS for Windows Version 12.0.

## RESULTS

The study sample consisted of 70 patients, 38 males and 32 females with a mean age of 34.6 years (SD 10.2). The mean duration of illness was 9.2 years (SD 6.2) with a mean frequency of 1.6 previous hospitalizations (SD 1.1). The mean chlorpromazine equivalent during admission or assessment was 468 mg (SD 416.5) as depicted in Table 1. Also the assessment of psychopathology by the Positive and Negative Symptom Scale (PANSS) revealed PANSS-positive mean score of 19.2 (SD 6.2), PANSS-negative mean score of 15 SD (7.4) and PANSS-general mean score of 38.0 (SD 7.8) [Table 1].

**Table 1:** Clinical characteristics of the sample and psychopathology

Mean age of sample	34.6 years (SD 10.2)
Mean duration of illness	9.2 years (SD 6.2)
Insidious onset of illness	52 (65%) patients
Cenesthesias present at onset	32 (40%) patients
Mean frequency of previous hospitalizations	1.6 (SD 1.1)
Chlorpromazine equivalent doses	468 mg (SD 416.5)
PANSS-positive mean score	19.2 (SD 6.2)
PANSS-negative mean score	15 (SD 7.4)
PANSS-general mean score	38.0 (SD 7.8)

The cenesthesias as assessed on the BSABS are summarized in Table 2. The most frequent bodily sensations reported included depersonalization, motor weakness, abnormal pain,‘numbness and stiffness’, ‘emptiness, heaviness, lightness, falling/sinking, levitation/elevation’ and ‘diminution, shrinking, enlargement, constriction’, each rated by more than 25% of the sample [Table 2].

**Table 2:** Scores on Bonn scale for the assessment of basic symptoms category D/cenesthesias

Cenesthesias	% of sample *n* = 70
Sensations of numbness, stiffness and feeling strange	34.2
Somatic depersonalization	55.7
Sensations of motor weakness	32.9
Sensations of pain	28.7
Migrating sensations	10.0
Electric sensation	20.0
Thermic sensation	21.4
Sensations of movement, pulling or pressure inside the body or on its surface	10.0
Sensations of abnormal heaviness, lightness or emptiness, of falling or sinking, levitation or elevation	44.2
Sensations of extension, diminution, shrinking, enlargement or constriction	25.7
Kinaesthetic sensations (feeling as if body moves without movement being observed)	5.7
Vestibular sensations	12.8
Dysaesthesia caused by physical contact	17.1
Unclassified cenesthesias	11.4
Dysesthetic crisis	1.43
Paroxysmic anxiety associated with symptoms	12.8

Assessment of the mean scores of body image aberration of the whole sample at admission and after 2 weeks, as presented in Table 3, indicate a tendency to underestimate lower extremities and feelings of body size change. Body cathexis was positive [Table 3].

**Table 3:** Body experience characteristics at baseline and at two weeks

	Sample *n* = 70 at baseline		Sample *n* = 64 at 2 weeks follow up assesment	
		
	Mean	SD	Mean	SD
BSABS cenesthesias	3.7	3.2	2.8	3.2
BPI-legs*	91.5	33.1	97.3	42.0
VAS small†	2.2	3.6	1.2	2.1
VAS large	2.1	3.5	1.7	3.5
VAS body size change	1.7	2.2	1.3	2.7
VAS body cathexis	5.9	2.8	6.3	3.3

* BPI = Body perception indices

† VAS = Visual analogue scale

The follow up assessment of 64 (91.4%) patients after treatment for 2 weeks revealed that there was partial improvement of the cenesthesias along with the body image. The association of the above symptoms did not show any significant association (Pearson's *r* or *t*-tests) with age, sex, number of previous hospitalizations, duration of illness, dosages of neuroleptic medication in chlorpromazine equivalents, depressive symptoms and extrapyramidal side-effects. With regard to psychopathological symptoms VAS large was positively correlated with PANSS-scores general and positive domains and also with scores of delusions (r = 0.32, *P* < 0.001), unusual thought content (r = 0.26, *P* < 0.05) and with grandiosity. A negative association between underestimation of lower extremities and anxiety (r = -0.28, *P* < 0.05) was found.

Cenesthesias were found to be positively correlated with disturbances of body concept, i.e. feeling as if the body or its parts feel unusually small (r = 0.45) and unusually large (r = 0.52) and as if the size of the body has changed (r = 0.42). But not with disturbances of body size perception (underestimation of lower extremities) and body cathexis.

The medical records of 57 (81.4%) of the patients were available and evaluated in detail. Also the patient and family members were interviewed in detail regarding the cenesthesias. In 88.8% of the patients two or more cenesthesias had been present in the past. They were noted to be present during all phases of the illness even when other symptomatology was minimal or the patient was on low doses of antipsychotic or off drugs. The cenesthesias also exacerbate or change in form when there is exacerbation of positive symptoms. A change in their nature i.e. changing from one form to another was noted in 75.7% of the patients.

## DISCUSSION

Patients with schizophrenia frequently report abnormal body sensations to clinicians. These sensations have also been described in the past. These descriptions have come from German, French and most importantly Russian psychiatrists who have more varied phenomenological tradition not so dependent upon Wernicke and Jasper. In anglo-merican literature, the experiences are most commonly described as hallucinations of bodily senses or somatic passivity. These varied descriptive terminologies have probably led to the patients with prominent cenesthesias being commonly diagnosed as paranoid schizophrenia and the diagnosis of cenesthopathic schizophrenia being rarely used or studied. The ICD-10 and no classificatory system give any diagnostic criterion for the same. This is the first Indian study to have comprehensively examined cenesthesias and body image aberration along with common psychopathology in a sample of paranoid schizophrenia. Despite the methodological limitations of the study such as the small sample size and the restriction to paranoid schizophrenia, many important results need discussion and emphasis. It must be emphasized that number of studies studying the phenomenology and its relationship are scanty. Moreover the inconsistency of symptom definitions, inclusion criteria and methodological approaches of these publications makes the comparison or drawing conclusions difficult.

The results support the repeated made observations of these cenesthesias being commonly present and reported by schizophrenia patients and being cause of their concern. Many of the sensations of pain, numbness, stiffness and feeling strange, abnormal heaviness, lightness, extension, diminution, shrinking, and enlargement of limbs were reported in more than 25% of the sample. Similar observations have also been reported by Braunig *et al.*,[16] but can also represent the characteristics of this sample. 40% of the patients were reported to have cenesthesias at or prior to the onset of the psychotic phase. Abnormal bodily sensations have also be included in the concept of ‘basic symptoms’ which have been found to be early predictors for developing schizophrenia.[17,18]

Assessing the relationship of different categories of abnormal bodily sensations, cenesthesias were found to be significantly positively related with disturbances of body concept. This suggests that probably these phenomena are misinterpretation of internal perception rather than perceptual aberration. There is also a possibility that both of them may be influencing each other or may be causally related. Huber[19] has suggested that the limbic system, diencephalic and parietal areas of the brain may have etiological importance in the genesis of cenesthesias. Many of these areas have been associated with disorders of body image and concept and also with perceptual disorders and schizophrenia. This group of patients has never been separately studied using functional imaging like hallucinations and delusions in schizophrenia. Further studies are needed to clarify their position along Anglo American or the germanophone and french conceptualization.

An improvement in indices of cenesthesia, body image and concept was noted in Table 3 of our study. Similar improvement have also been reported in the past by Huber[20] and Smulevich *et al.*[21] The review of the patient records revealed that 88.8% of the patients two or more cenesthesias and that in 75.7% patients a change from one form to another was noted. They were noted to be present during all phases of the illness Similar results have been reported in the past by Huber[19] and Kato.[22] According to them the course of these patients are characterized by changing nature of their cenesthesias which persist only for short duration and then change into another form, but are present during substantial course of the illness.

Clinical implications of identifying a subgroup with marked body related symptomatology remain to be explored in further longitudinal studies. Abnormal bodily sensations are also represented within the concept of ‘basic symptoms’ which has recently been found to be early predictors for developing schizophrenia.[17,18] The status of these symptoms in the current domains of psychopathology of schizophrenia also needs further evaluation. The patients with subtypes other than paranoid schizophrenia have to be investigated in future to assess whether a similar subgroup with abnormal bodily sensations can be identified across the spectrum of subtypes.
